# Supplementary material for: Ciliary transition zone proteins coordinate ciliary protein composition and ectosome shedding
Source: Nat Commun. 2022 Jul 9;13:3997. doi: 10.1038/s41467-022-31751-0 (PMC9271036; doi:10.1038/s41467-022-31751-0)
Supplement: Supplementary file 1 — Supplementary Information [file 41467_2022_31751_MOESM1_ESM.pdf]

Ciliary transition zone proteins coordinate ciliary protein composition and ectosome shedding

Liang Wang[1, #, *], Xin Wen[1, #], Zhengmao Wang[2,3, #], Zaisheng Lin[4], Chunhong Li[1], Huilin Zhou[1], Huimin Yu[1], Yuhan Li[1], Yifei Cheng[1], Yuling Chen[5], Geer Lou[6], Junmin Pan[2,3], Muqing Cao[4, *]

[1]School of Life Sciences, Jiangsu Normal University, Xuzhou 221116, China

[2]MOE Key Laboratory of Protein Sciences, School of Life Sciences, Tsinghua University, Beijing 100084, China

[3]Laboratory for Marine Biology and Biotechnology, Qingdao National Laboratory for Marine Science and Technology, Qingdao 266071, China

[4]Key Laboratory of Cell Differentiation and Apoptosis of Chinese Ministry of Education, Department of Pathophysiology, Shanghai Jiao Tong University School of Medicine, Shanghai 200025, China

[5]School of Life Sciences, Tsinghua University, Beijing 100084, China

[6]Shanghai Biotree Biotech Co. Ltd, Shanghai 201815, China

[#]L.W., X.W., and Z.W. contributed equally to this work.

[*]To whom correspondence may be addressed. **Email:** wangliang@jsnu.edu.cn, muqingcao@sjtu.edu.cn.

Keywords: cilia; transition zone; TCTN1; ectosome

This PDF file includes:

Supplementary Figures 1 to 7

Supplementary Figure 1. TCTN1 is localized at the TZ.

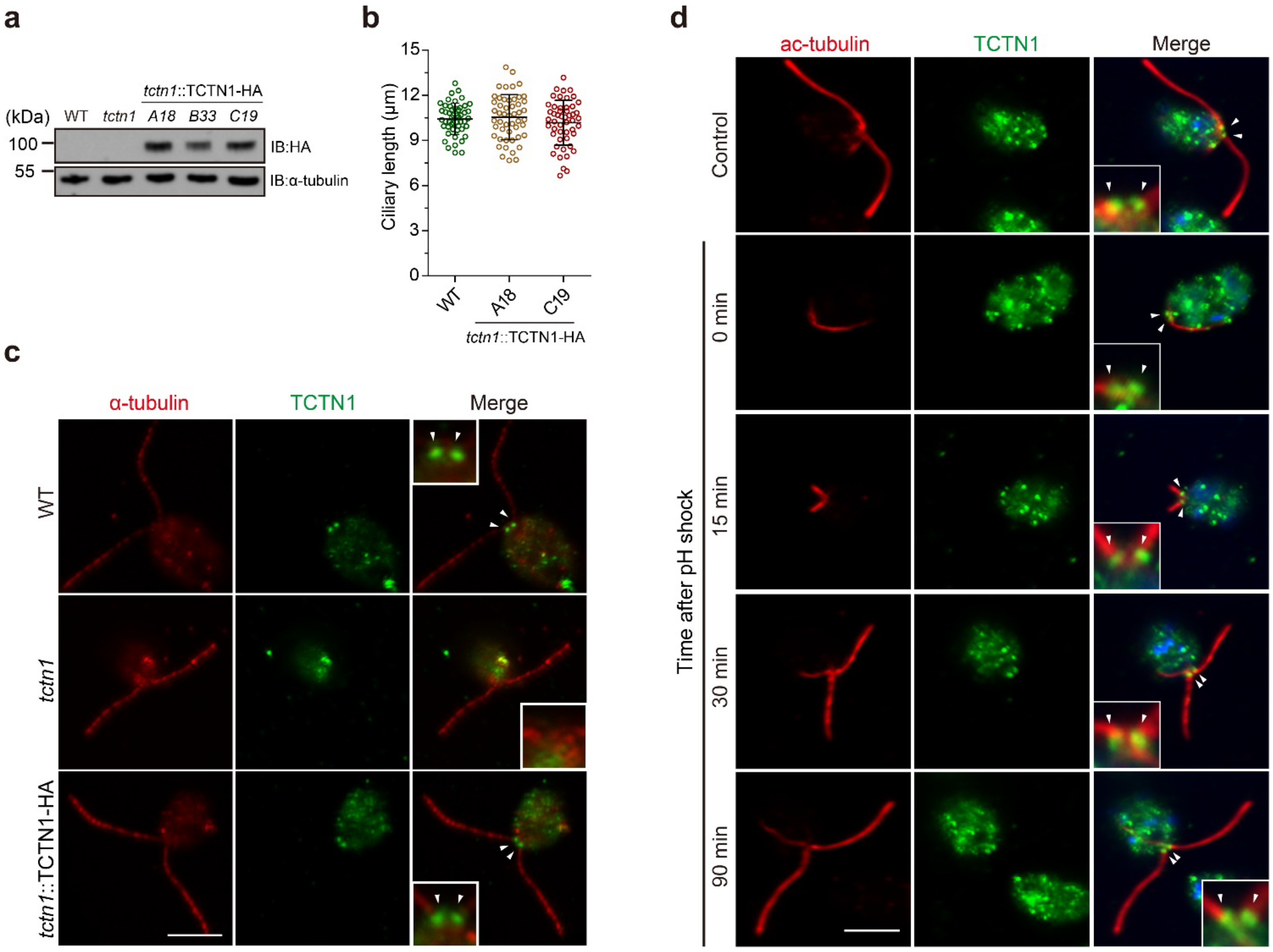

a Immunoblot analysis of the expression of TCTN1-HA in the WT, *tctn1*, and rescued cells. Cell lysates from the indicated cells were subjected to immunoblotting probed with the indicated antibodies. WT and *tctn1* cells were used as negative controls. α-tubulin was used as a loading control. The positions of standard proteins and their molecular masses in kDa are indicated. **b** Scatter plot showing the mean ± SD of ciliary length (n=50) in the WT, *tctn1*, and rescued cells (*A18* and *C19*). **c** Immunostaining images depicting the immunospecificity of the anti-TCTN1 antibody and localization of TCTN1 in the TZ. WT, *tctn1*, and the rescued cells expressing TCTN1-HA were immunostained with anti-TCTN1 (green) and anti-α-tubulin (red) antibodies. The arrowheads indicate the TZ at the ciliary base. The insets show the higher magnification views of the TZ region in the merge image. Scale bar, 5 μm. **d** Immunostaining images illustrating that the localization of TCTN1 in the TZ was not changed during ciliary regeneration. Cells were subjected to pH shock to induce deciliation and collected for immunostaining with anti-TCTN1 (green) and anti-acetylated α-tubulin (ac-tubulin, red) antibodies at the indicated time point. The nucleus was stained with DAPI (blue). The arrowheads indicate the TZ at the ciliary base. The insets show higher magnification views of the TZ region in the merged image. Scale bar, 5 μm. Source data are provided as a Source Data file.

Supplementary Figure 2. The ultra-structural localization of TCTN1.

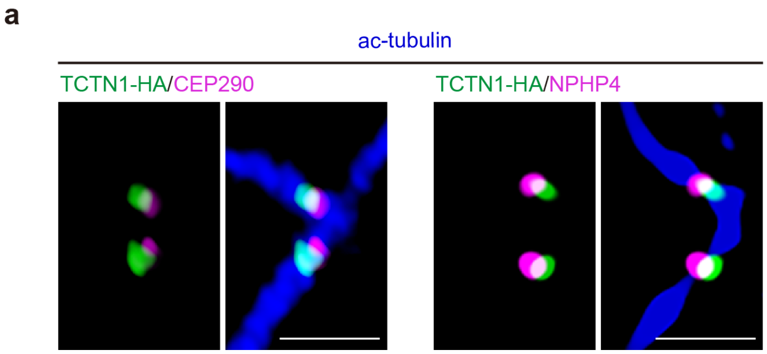

a Super-resolution imaging showing the localization of TCTN1-HA and CEP290/NPHP4. The rescued cells expressing TCTN1-HA were immunostained with anti-acetylated α-tubulin (ac-tubulin, blue), anti-HA (green), and anti-CEP290 (magenta) or anti-NPHP4 (magenta) antibodies. Scale bar, 1 μm.

Supplementary Figure 3. The ciliary phenotypes of *tctn1*, *cep290*, and *nphp4* are different.

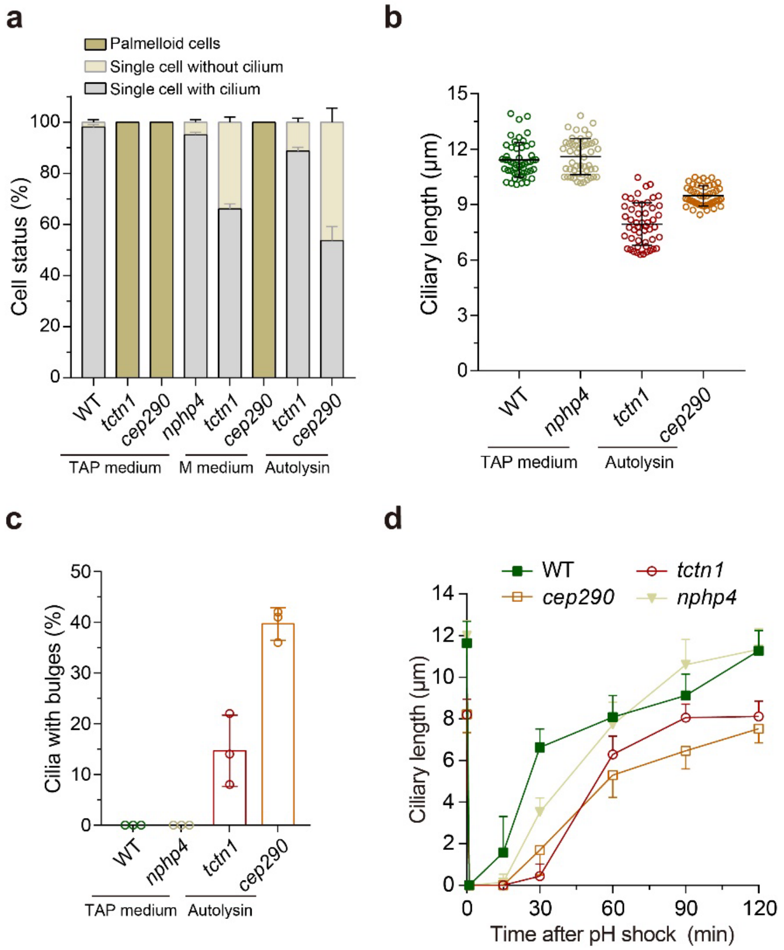

a Column graph showing the cell status (%) under different conditions (TAP medium, M medium, or after autolysin treatment) for WT, *tctn1*, *cep290*, and *nphp4* cells. **b** Scatter plot showing the average ciliary length under different conditions (TAP medium or after autolysin treatment) for WT, *tctn1*, *cep290*, and *nphp4* cells. **c** Bar–scatter graph summarizing the percentages of cilia with bulges (%) in WT, *tctn1*, *cep290*, and *nphp4* cells. The palmelloid cells (*tctn1* and *cep290*) were treated with autolysin for hatching before bulge counting. **d** Ciliary regeneration after deciliation by pH shock of WT, *tctn1*, *cep290*, and *nphp4* cells. The palmelloid cells (*tctn1* and *cep290*) were treated with autolysin and grown for 3 h before the deciliation assay and ciliary length analysis. Data are shown as the mean ± SD (*n*=50 for the ciliary length; *n*=200 for the cell numbers) and repeated three times in this figure. Source data are provided as a Source Data file.

Supplementary Figure 4. Enrichment of IFT particles in the *tctn1* mutant.

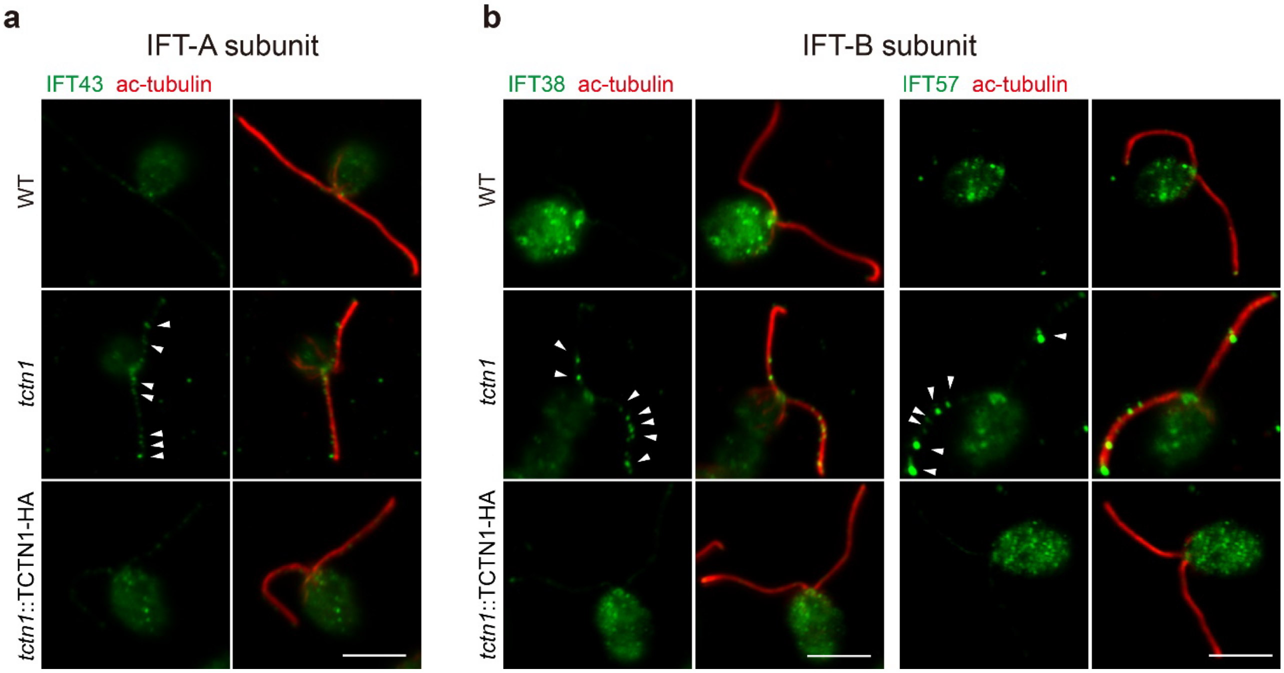

a, b Immunostaining images displaying the enrichment of IFT subunits in *tctn1* cells. WT, *tctn1*, and rescued cells were immunostained with anti-IFT43 (**a**, green), or anti-IFT38 (**b**, left, green), or anti-IFT57 (**b**, right, green), and anti-acetylated α-tubulin (ac-tubulin, red) antibodies. The arrowheads indicate the accumulation of IFT particles in the cilium. Scale bar, 5 μm.

Supplementary Figure 5. PSBC and PSAD staining reveal cup-shaped chloroplasts.

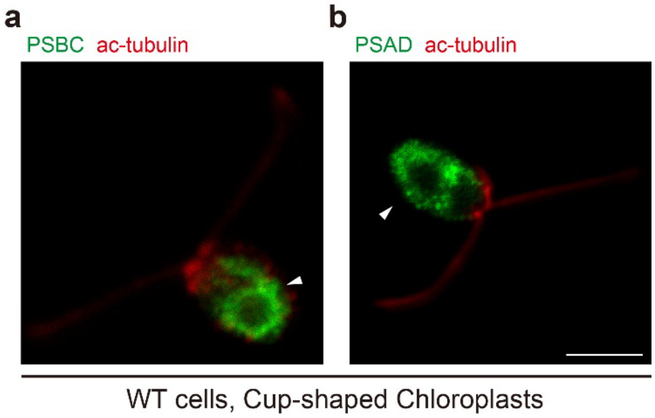

a, b Immunofluorescence images demonstrating the validity of antibodies against the photosystem proteins PSBC and PSAD in the chloroplast of WT cells. WT cells were immunostained with anti-PSBC (**a**, green), or anti-PSAD (**b**, green), and anti-acetylated α-tubulin (ac-tubulin, red) antibodies. The arrowheads show the cup-shaped chloroplasts. Scale bar, 5 μm.

Supplementary Figure 6. The misregulated proteins in different TZ mutants (*tctn1*, *cep290*, and *nphp4*).

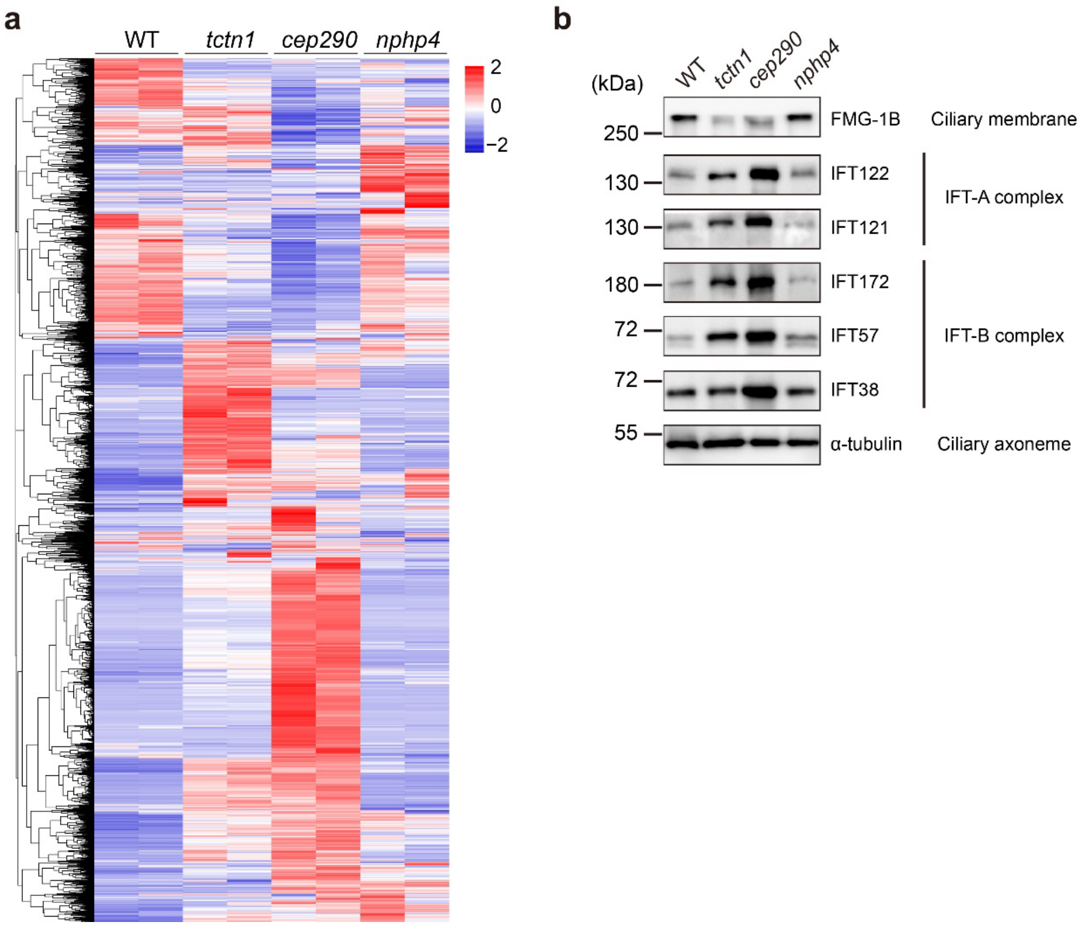

a Heatmap of all 2845 proteins identified in cilia from WT, *tctn1*, *cep290*, and *nphp4*. Scaled abundance in the expression of proteins from two biological replicates of these four strains are normalized Z-scores and displayed as colors ranging from red (upregulated) to blue (downregulated). **b** Immunoblot of cilia isolated from WT, *tctn1*, *cep290*, and *nphp4* cells. Antibodies against FMG1-B, IFT-A complex (IFT121 and IFT122), and IFT-B complex (IFT38, IFT57, and IFT172) were used to confirm the proteomics results shown in **a**. α-tubulin was used as a loading control. The positions of standard proteins and their molecular masses in kDa are indicated. Source data are provided as a Source Data file.

Supplementary Figure 7. The protein composition of the ciliary ectosomes shedding from gametes of different TZ mutants are different.

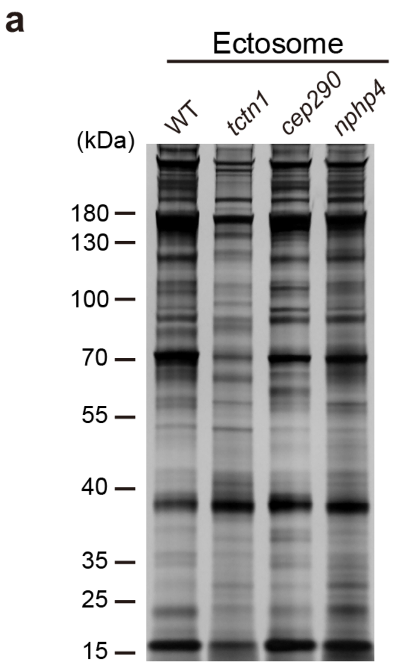

a Silver-stained SDS–PAGE gel showing protein variations in ectosomes isolated from the plus mating type gamete (21*gr*, *tctn1*, *cep290*, *nphp4*) with minus mating type gamete (6145c). The indicated ciliary ectosomes purified from the same amount of cells were loaded with equal amount of proteins, separated on 4% ~ 12% SDS–PAGE and then visualized by silver staining. Source data are provided as a Source Data file.
