# Supplementary material for: Ciliary transition zone proteins coordinate ciliary protein composition and ectosome shedding
Source: Nat Commun. 2022 Jul 9;13:3997. doi: 10.1038/s41467-022-31751-0 (PMC9271036; doi:10.1038/s41467-022-31751-0)
Supplement: Supplementary file 3 — Reporting Summary [file 41467_2022_31751_MOESM3_ESM.pdf]

nature portfolio

Corresponding author(s): Liang Wang, Muqing Cao

Last updated by author(s): Jun 28, 2022

Reporting Summary

Nature Portfolio wishes to improve the reproducibility of the work that we publish. This form provides structure for consistency and transparency in reporting. For further information on Nature Portfolio policies, see our Editorial Policies and the Editorial Policy Checklist.

Statistics

For all statistical analyses, confirm that the following items are present in the figure legend, table legend, main text, or Methods section.

n/a	Confirmed	
☐	☒	The exact sample size (n) for each experimental group/condition, given as a discrete number and unit of measurement
☐	☒	A statement on whether measurements were taken from distinct samples or whether the same sample was measured repeatedly
☐	☒	The statistical test(s) used AND whether they are one- or two-sided<br>*Only common tests should be described solely by name; describe more complex techniques in the Methods section.*
☒	☐	A description of all covariates tested
☐	☒	A description of any assumptions or corrections, such as tests of normality and adjustment for multiple comparisons
☐	☒	A full description of the statistical parameters including central tendency (e.g. means) or other basic estimates (e.g. regression coefficient) AND variation (e.g. standard deviation) or associated estimates of uncertainty (e.g. confidence intervals)
☐	☒	For null hypothesis testing, the test statistic (e.g. F, t, r) with confidence intervals, effect sizes, degrees of freedom and P value noted<br>*Give P values as exact values whenever suitable.*
☒	☐	For Bayesian analysis, information on the choice of priors and Markov chain Monte Carlo settings
☒	☐	For hierarchical and complex designs, identification of the appropriate level for tests and full reporting of outcomes
☒	☐	Estimates of effect sizes (e.g. Cohen's d, Pearson's r), indicating how they were calculated

Our web collection on statistics for biologists contains articles on many of the points above.

Software and code

Policy information about availability of computer code

Data collection	Leica Application Suite imaging software (LAS version 4.0.0); Leica Application Suite X (version 3.1.5.16308); NIS-Elements AR (version 5.31); Amersham imager 600 Control (version 1.2.0)
Data analysis	ImageJ (version 1.49V); GraphPad Prism (version 7.00); Adobe Illustrator CC (version 18.1.0); Adobe Photoshop CC (version 14.0); Proteome Discoverer software (PD, version 2.4)

For manuscripts utilizing custom algorithms or software that are central to the research but not yet described in published literature, software must be made available to editors and reviewers. We strongly encourage code deposition in a community repository (e.g. GitHub). See the Nature Portfolio guidelines for submitting code & software for further information.

Data

Policy information about availability of data

All manuscripts must include a data availability statement. This statement should provide the following information, where applicable:

- Accession codes, unique identifiers, or web links for publicly available datasets
- A description of any restrictions on data availability
- For clinical datasets or third party data, please ensure that the statement adheres to our policy

The mass spectrometry proteomics data have been deposited to the ProteomeXchange Consortium via the PRIDE partner repository with the dataset identifier PXD033090. The accessible hyperlink of Chlamydomonas reinhardtii v5.6 genome database is https://phytozome-next.jgi.doe.gov/info/Creinhardtii_v5_6. All other raw data that support the findings of this study are available from the corresponding author upon reasonable request. Source data are provided with this paper.

Field-specific reporting

Please select the one below that is the best fit for your research. If you are not sure, read the appropriate sections before making your selection.

☒ Life sciences ☐ Behavioural & social sciences ☐ Ecological, evolutionary & environmental sciences

For a reference copy of the document with all sections, see nature.com/documents/nr-reporting-summary-flat.pdf

Life sciences study design

All studies must disclose on these points even when the disclosure is negative.

Sample size	Sample sizes were determined according to the related references (e.g. PMID: 31371489; PMID: 23970417) and chosen based on logistical and experimental experiences. In every case, three or two biologiclal replicates for each experiment were included. All experiments were reliably reproduced and the number of samples chosen was sufficient to support meaningful conclusions.
Data exclusions	No data exclusions were performed. Outlines were identified, but retained in analysis.
Replication	Multiple biological replicates were used as indicated in the Methods or Figure Legends, and all experiments were reliably reproduced.
Randomization	All transgenic algal clones used in this study were picked randomly with passage cultivation. Inoculation, cultivating, harvesting, processing and imaging of samples was performed randomly into different treatment groups for each biological replicate.
Blinding	Blinding was not applicable to this study as the data grouping and analysis were performed in an unbiased manner. Data was analyzed with common strategies.

Reporting for specific materials, systems and methods

We require information from authors about some types of materials, experimental systems and methods used in many studies. Here, indicate whether each material, system or method listed is relevant to your study. If you are not sure if a list item applies to your research, read the appropriate section before selecting a response.

Materials & experimental systems

n/a	Involved in the study
☐	☒ Antibodies
☐	☒ Eukaryotic cell lines
☒	☐ Palaeontology and archaeology
☒	☐ Animals and other organisms
☒	☐ Human research participants
☒	☐ Clinical data
☒	☐ Dual use research of concern

Methods

n/a	Involved in the study
☒	☐ ChIP-seq
☒	☐ Flow cytometry
☒	☐ MRI-based neuroimaging

Antibodies

Antibodies used	The primary antibodies used for immunoblotting (IB) or immunofluorescence (IF) were as follows: anti-HA high affinity (clone 3F10, rat monoclonal IgG, 1:3000 for IB and 1:50 for IF; Cat. No. 11867423001, Roche, Switzerland), anti-α-tubulin (clone 1E4C11, mouse monoclonal IgG, 1:5000 for IB and 1:200 for IF; Cat. No. 66031-1-Ig, Proteintech, USA), anti-α-tubulin (rabbit polyclonal IgG, 1:5000 for IB; Cat. No. 11224-1-AP, Proteintech, USA), anti-centrin (clone 20H5, mouse monoclonal IgG, 1:400 for IF; Cat. No. 04-1624, Merck Millipore, Germany), anti-acetylated α-tubulin (clone 6-11B-1, mouse monoclonal IgG, 1:200 for IF; Cat. No. T7451, Sigma, USA), anti-CEP290 (rabbit polyclonal IgG, 1:200 for IF; a gift from Dr. George Witman), anti-NPHP4 (rabbit polyclonal IgG, 1:100 for IF; a gift from Dr. George Witman), anti-FMG-1B (clone SP2/0, mouse monoclonal IgG, 1:100 for IB; Cat. No. AB_2722112, Developmental Studies Hybridoma Bank), anti-IFT122 (rabbit polyclonal IgG, 1:2000 for IB and 1:100 for IF), anti-IFT121 (rabbit polyclonal serum, 1:2000 for IB), anti-IFT172 (rabbit polyclonal IgG, 1:2000 for IB), anti-IFT57 (rabbit polyclonal IgG, 1:2000 for IB and 1:100 for IF), anti-IFT54 (rabbit polyclonal serum, 1:2000 for IB), anti-IFT43 (rabbit polyclonal serum, 1:100 for IF), anti-IFT38 (rabbit polyclonal IgG, 1:5000 for IB and 1:100 for IF), anti-FLA10 (rabbit polyclonal IgG, 1:3000 for IB), anti-D1BLIC (rabbit polyclonal IgG, 1:2000 for IB), anti-IC2/IC69 (clone 1869A, mouse monoclonal IgG, 1:20000 for IB; Cat. No. D6168, Sigma, USA), anti-PSAD (rabbit polyclonal serum, 1:1000 for IB, 1:500 for IF; a gift from Dr. Xiaobo Li; Cat. No. AS09461, Agrisera, Sweden), and anti-PSBC (rabbit polyclonal serum, 1:3000 for IB, 1:500 for IF; a gift from Dr. Xiaobo Li; Cat. No. AS111787, Agrisera, Sweden). Rabbit anti-RIB72 antibodies were generated against bacterial expressed GST-His-tagged RIB72 (25–290 amino acids) (Abclonal, China) and were only used at a dilution of 1:3000 for IB. Rabbit anti-BBS8 antibodies were generated against bacterial expressed GST-His-tagged BBS8 (16–193 amino acids) (Abclonal, China) and were only used at a dilution of 1:250 for IB. Rabbit anti-TCTN1 antibodies were generated against bacterial expressed GST-His-tagged TCTN1 (368–490 amino acids) (Abclonal, China) and were only used at a dilution of 1:500 for IF.<br>The secondary antibodies used for WB were HRP-conjugated goat anti-mouse, goat anti-rabbit and goat anti-rat (1:5000; Cat. No. 115-035-003, Cat. No. 111-035-003, Cat. No. 112-035-003, Jackson, USA). The secondary antibodies used for IF were preadsorbed

anti-rat IgG H&L (Alexa Fluor 488) (Cat. No. ab150165), preadsorbed anti-mouse IgG H&L (Alexa Fluor 594) (Cat. No. ab150120), preadsorbed anti-rabbit IgG H&L (Alexa Fluor 647) (Cat. No. ab150087), preadsorbed anti-rabbit IgG H&L (Alexa Fluor 594) (Cat. No. ab150084), preadsorbed anti-rabbit IgG H&L (Alexa Fluor 488) (Cat. No. ab150077), and preadsorbed anti-mouse IgG H&L (Alexa Fluor 647) (Cat. No. ab150119) (Abcam, UK). The secondary antibodies were used at a dilution of 1:500.
Anti-HA (rabbit monoclonal antibody, clone C29F4, Cat. No. 3724, CST, USA) was only used at a dilution of 1:10 for immunoEM. Goat anti-Rabbit gold-conjugated secondary antibody (goat polyclonal antibodies, 10 nm, Cat. No. G7402, Sigma, USA) was only used at a dilution of 1:10 for immunoEM.

Validation

The anti-TCTN1 antibodies were generated against GST-His-tagged TCTN1 (368–490 amino acids) in this study (Abclonal, https://abclonal.com.cn/polyclonal-antibody/). We performed Westernblot and IF experiments in WT and tctn1 mutant cells to confirm the specificity of the polyclonal antibodies. The anti-RIB72 antibodies and anti-BBS8 antibodies were generated against GST-His-tagged RIB72 (25–290 amino acids) or GST-His-tagged BBS8 (16–193 amino acids) in this study (Abclonal, https://abclonal.com.cn/polyclonal-antibody/). We performed Westernblot experiments in WT and mutant cells to confirm the specificity of the polyclonal antibodies.
For other antibodies, we used the antibodis that had been validated in other published studies or by the commercial companies.
anti-HA high affinity: https://www.sigmaaldrich.cn/CN/zh/product/roche/roahaha?gclid=CjwKCAjwxZqSBhAHEiwASr9n9CqUb1mhr9AxkgunLSv_srosrFau7EFPbbtlFq3GFyfl1gBDdTqPlxoCBQ0QAvD_BwE
anti-α-tubulin: https://www.ptglab.com/products/tubulin-Alpha-Antibody-66031-1-Ig.htm
anti-α-tubulin: https://www.ptglab.com/products/TUBA1B-Antibody-11224-1-AP.htm
anti-centrin: https://www.sigmaaldrich.cn/CN/zh/product/mm/041624?gclid=CjwKCAjwxZqSBhAHEiwASr9n9Lj7gp2KqGV5bHEGbAy-jLd1kOhzy0-cEIrkC-vvmjvYYeVxN55ltxoCwAkQAvD_BwE
anti-acetylated α-tubulin: https://www.sigmaaldrich.cn/CN/zh/product/sigma/t6793?gclid=CjwKCAjwxZqSBhAHEiwASr9n9LrUu8Oyzl4lszw7OkW34Ws5rGN1czk23AhbMXCyYJHhCByQHjevIBoCvvYQAvD_BwE
anti-CEP290, anti-NPHP4: a gift from Dr. George Witman, (reference: PMID: 20819941, PMID: 25150219)
anti-FMG-1B: https://dshb.biology.uiowa.edu/FMG-1B-61
anti-HA (CST): https://www.cellsignal.cn/products/primary-antibodies/ha-tag-c29f4-rabbit-mab/3724?site-search-type=Products&N=4294956287&Ntt=ha&fromPage=plp
anti-IFT121, anti-IFT172, anti-IFT38, anti-FLA10, anti-D1BLIC, anti-IC2/IC69: reported in PMID: 33368450
anti-IFT122, anti-IFT54, anti-IFT43,: reported in PMID: 28207750
anti-IFT57: reported in PMID: 33112235
anti-PSAD: https://www.agrisera.com/en/artiklar/psad-psi-d-subunit-of-photosystem-i-2.html
anti-PSBC: https://www.agrisera.com/en/artiklar/psbc-cp43-protein-of-psii-2.html
anti-Rabbit gold-conjugated secondary antibody: https://www.sigmaaldrich.cn/CN/zh/product/sigma/g7402

Eukaryotic cell lines

Policy information about cell lines

Cell line source(s)

21gr (CC-1690, wild-type, mt+), 6145c (CC-2895, wild-type, mt-), cep290 (CC-4374, mt+), and nphp4 (CC-5113, mt+) were prvided by the Chlamydomonas Resource Center (https://www.chlamycollection.org/). tctn1 mutant cell line was obtained in this work by screening the mutant library.

Authentication

Authentication of the Chlamydomonas cells were based on the phenotypic analysis on plates and in liquid culture. PCR confirmation was also performed. The mating types of WT cells (21gr or 6145c) were authenticated via gamate mating. Mutants (cep290, nphp4, and tctn1) were authenticated via IF with conrresponding antibodies.

Mycoplasma contamination

It is not applicable to this work.

Commonly misidentified lines
(See ICLAC register)

No commonly misidentified lines were used in this study.
